# Prevalence of Vitamin B12 Deficiency in Type 2 Diabetic Patients on Long-Term Metformin Therapy

**DOI:** 10.7759/cureus.88265

**Published:** 2025-07-18

**Authors:** Felicita M Tayong, Timothy Oluwabamise Francis, Jestin KJ, Raghabendra Kumar Mahato, Hayat Ullah, Sidra Kamal, Khurshid Kamil

**Affiliations:** 1 General Surgery, Tulane University School of Medicine, New Orleans, USA; 2 College of Medicine, University of Science, Arts and Technology, British West Indies, MSR; 3 Medicine, All Saints University School of Medicine, Roseau, DMA; 4 Pathology and Laboratory Medicine, All India Institute of Medical Sciences, Bilaspur, IND; 5 Medicine, Gandaki Medical College Teaching Hospital and Research Center, Pokhara, NPL; 6 Medicine, St Hugh's Hospital, Peshawar, PAK; 7 Emergency Medicine, Aga Khan University Hospital, Karachi, PAK; 8 College of Medicine, Karachi Medical and Dental College, Karachi, PAK; 9 Medicine, Lady Reading Hospital, Peshawar, PAK

**Keywords:** metformin, prevalence, risk factors, type 2 diabetes mellitus, vitamin b12 deficiency

## Abstract

Introduction: Vitamin B12 deficiency is a recognized complication in patients with type 2 diabetes mellitus (T2DM) undergoing long-term metformin therapy. Despite metformin’s widespread use due to its efficacy and safety profile, it has been associated with reduced absorption of vitamin B12, which can lead to hematologic and neurologic complications. This study aimed to determine the prevalence of vitamin B12 deficiency and its associated risk factors among T2DM patients on prolonged metformin therapy in Peshawar, Pakistan.

Methodology: A descriptive cross-sectional study was conducted in the outpatient departments of tertiary care hospitals in Peshawar from October 2024 to April 2025. A total of 320 T2DM patients aged 18 years and above who had been on metformin therapy were included. Data were collected through structured questionnaires and medical records, and serum vitamin B12 levels were measured using blood samples. Deficiency was defined as <200 pg/mL, borderline as 200-300 pg/mL, and normal as >300 pg/mL. Data were analyzed using SPSS version 20 (IBM Corp., Armonk, NY, US), applying chi-square tests to assess associations.

Results: Out of 329 participants, 65.7% (n=216) had vitamin B12 deficiency, 24.3% (n=80) had borderline levels, and only 10% (n=33) had normal vitamin B12 levels. A statistically significant association was found between vitamin B12 deficiency and increasing age (p=0.003), higher BMI (p=0.012), longer duration of diabetes (p=0.025), and prolonged metformin use (p<0.001).

Conclusion: The study reveals a high prevalence of vitamin B12 deficiency among T2DM patients on long-term metformin therapy. The deficiency is significantly associated with older age, obesity, longer diabetes duration, and extended metformin use. Routine screening and early supplementation of vitamin B12 should be considered in diabetic patients on prolonged metformin treatment to prevent potential complications.

## Introduction

Type 2 diabetes mellitus (T2DM) is one of the most common and debilitating non-communicable illnesses in the world. Its pathogenesis includes both genetic and environmental factors [1]. Controlling blood glucose levels within the desired range is the primary goal of T2DM treatment, aiming to prevent complications that can reduce the patient's quality of life and life expectancy [2]. The most prevalent kind of diabetes, T2DM, affects 90% to 95% of diabetics. Insulin resistance, a condition in which the body does not utilize insulin as it should, and decreased insulin production are the two primary symptoms of T2DM. Genetic factors, a family history of diabetes, advanced age, obesity, and a sedentary lifestyle are examples of this sort of risk factor [3].

One medication that is frequently used to treat T2DM is metformin [4]. According to the majority of current international guidelines, such as those issued by the American Diabetes Association and the Korean Diabetes Association, when diabetes is first diagnosed, metformin should be the first oral medication prescribed (if tolerated and not contraindicated) in conjunction with lifestyle changes [5]. The medication works by increasing peripheral insulin sensitivity and decreasing hepatic glucose synthesis. Additionally, it might be useful as a treatment for polycystic ovarian disease and cardiovascular disorders [6]. Despite being widely used, affordable, and effective, metformin has several side effects that can reduce drug tolerance. Vitamin B12 deficiency is one of the most overlooked adverse effects of metformin. Although the exact mechanisms underlying metformin-induced vitamin B12 deficiency remain unclear, several plausible mechanisms have been put forth, including decreased intrinsic factor secretion, inhibition of absorption of the intrinsic factor-vitamin B12 complex, modifications to bile acid metabolism and reabsorption, and disruption of the intrinsic factor-vitamin B12 complex's ability to bind to the cubilin receptor [4,7]. Mostly found in animal foods such as meats, poultry, fish, eggs, and dairy products, vitamin B12 is a water-soluble vitamin that is essential for DNA synthesis, brain function, and healthy hematopoiesis. Therefore, megaloblastic anemia, gastrointestinal issues, and brain impairment are clinical signs of vitamin B12 insufficiency [8].

Numerous investigations have documented the link between vitamin B12 insufficiency and metformin use, with the first one being conducted in the early 1970s [9]. Additionally, an observational study showed that using metformin and proton pump inhibitors (PPIs) or histamine H2-antagonists at the same time increased the risk of having a vitamin B12 deficiency by 22% [10]. However, the incidence of metformin-induced vitamin B12 insufficiency ranges between locales and ethnicities; the percentage varies from 4.3% up to 30% [9]. In India's general population, vitamin B12 insufficiency rates range from 12% to 67% [11]. In support of this, a Pakistani case-control research revealed that 31% of patients receiving metformin had low serum B12 levels [12].

Metformin remains the first-line pharmacological treatment for T2DM due to its effectiveness in glycemic control and favorable safety profile. However, long-term use of metformin has been increasingly associated with vitamin B12 malabsorption, which may lead to hematological abnormalities and irreversible neurological damage if left undetected. In regions like Pakistan, where routine screening for micronutrient deficiencies is uncommon, there is a pressing need to assess the magnitude of this problem in diabetic patients. Despite metformin’s widespread use, limited local data exist on the prevalence and risk factors of vitamin B12 deficiency in the diabetic population of Peshawar. This study was, therefore, conducted to fill that knowledge gap, raise awareness among healthcare providers, and support the implementation of regular B12 screening protocols for patients on long-term metformin therapy, ultimately aiming to improve diabetic care and prevent associated complications.

## Materials and methods

This descriptive cross-sectional study was conducted at the Internal Medicine and Diabetic Outpatient Department of Lady Reading Hospital, Peshawar, Pakistan. The study duration spanned from October 2024 to April 2025.

Study population

The study population consisted of patients diagnosed with T2DM who were attending outpatient clinics and currently receiving metformin therapy.

Inclusion criteria

Participants were included in the study if they were aged 18 years or older, had a confirmed diagnosis of T2DM, had been on metformin therapy for at least six months (indicating long-term use), and provided written informed consent to participate.

Exclusion criteria

Participants were excluded if they had taken vitamin B12 supplements within the past six months, were strict vegetarians or vegans, had a history of bariatric or gastric surgery, were diagnosed with pernicious anemia, chronic alcoholism, or neurological disorders unrelated to diabetes, or were taking medications known to interfere with vitamin B12 levels, such as PPIs and H2 receptor blockers.

Sampling technique

A non-probability consecutive sampling technique was used. All eligible patients visiting the outpatient departments during the study period were invited to participate until the required sample size of 320 was achieved.

Data collection 

Data were collected using a structured questionnaire designed to obtain detailed information on participants’ demographics, clinical history, and medication use. Additionally, blood samples were taken from each participant to measure serum vitamin B12 levels. Long-term metformin use was defined as continuous use for six months or more. Vitamin B12 status was classified into three categories: deficient (<200 pg/mL), borderline (200-300 pg/mL), and normal (>300 pg/mL).

Data analysis

The data were entered and analyzed using SPSS version 20 (IBM Corp., Armonk, NY, US). Frequencies and percentages were calculated for categorical variables, while means and standard deviations were reported for continuous variables. The chi-square test was applied to examine associations between metformin dose or duration and vitamin B12 deficiency. Independent t-tests were used to compare mean values between different groups.

Ethical considerations

Approval for the study was obtained from the Institutional Review Board (IRB) of Lady Reading Hospital, Peshawar. All participants provided written informed consent, and confidentiality of data was strictly maintained throughout the study.

## Results

Table 1 presents the baseline sociodemographic and clinical characteristics of the 320 participants included in the study. The age distribution shows that the majority of participants (39%) were between 51 and 60 years of age, followed by 23.4% in the 40-50 age group, 20.6% aged over 60 years, and 16.8% in the 18-39 age group. The mean age of the participants was 54 years, indicating a predominantly middle-aged population. Regarding gender distribution, 60% of the participants were female, while 40% were male.

**Table 1:** Baseline sociodemographic and clinical characteristics of the study participants

Variables	Category	Frequency	Percentage	Mean
Age	18-39	54	16.8%	54
	40-50	75	23.4%	
	51-60	125	39%	
	More than 60	66	20.6%	
Gender	Male	128	40%	
	Female	192	60%	
BMI	Normal	32	10%	
	Preobese	96	30%	
	Obese	128	40%	
	Severe obese	64	20%	
Duration of diabetes in years	1 to 2 years	90	28.1%	
	3 to 5 years	164	51.2%	
	More than 6 years	66	20.7%	
Duration of metformin use in months/years	Less than 6 months	25	7.8%	
	6 to 12 months	100	31.2%	
	2 to 3 years	74	23.1%	
	More than 4 years	121	37.9%	
Vitamin B12 level	Normal (above 300 Pg/mL)	32	10%	
	Borderline (200 to 300 Pg/mL)	78	24.3%	
	Deficient (below 200 Pg/mL)	210	65.7%	

In terms of BMI, a significant portion of the population fell into the obese category (40%), with an additional 30% being preobese, 20% severely obese, and only 10% having a normal BMI. This suggests a high prevalence of overweight and obesity among the study population. The duration of diabetes varied, with more than half of the participants (51.2%) having lived with diabetes for three to five years, while 28.1% had diabetes for one to two years, and 20.7% for more than six years.

When looking at the duration of metformin use, 37.9% of participants had been on the medication for more than four years, 31.2% for six to 12 months, 23.1% for two to three years, and a smaller group of 7.8% had used it for less than six months. The data on vitamin B12 levels revealed that a large majority of the participants (65.7%) were deficient, while 24.3% had borderline levels and only 10% had normal B12 levels. This indicates a potential association between long-term metformin use and vitamin B12 deficiency, which may warrant further clinical attention (Table 1).

Table 2 presents a significant association between vitamin B12 levels and variables such as age, BMI, and duration of diabetes. The chi-square test and p-values indicate that these associations are statistically significant, as all p-values are less than 0.05. Among different age groups, the prevalence of vitamin B12 deficiency increases markedly with age. In the 18-39 age group, 62.9% had normal levels while only 13.1% were deficient. However, in individuals aged above 60 years, only 9% had normal levels, whereas a substantial 76% were deficient. This trend clearly shows that vitamin B12 deficiency becomes more prevalent as age increases (p=0.001).

**Table 2:** Association of vitamin B12 levels with age, BMI, and duration of diabetes in patients with type 2 diabetes mellitus

Variables	Category	Vitamin B12 level			P-value	Chi-square value
		Normal (above 300 Pg/mL), N (%)	Borderline (200 to 300 Pg/mL), N (%)	Deficient (below 200 Pg/mL), N (%)`		
Age	18-39	34 (62.9%)	13 (24%)	7 (13.1%)	0.001	640.00
	40-50	40 (43.1%)	20 (26.6%)	15 (20%)	0.001	532.00
	51-60	29 (23.2%)	30 (24%)	66 (52.8%)	0.001	690.00
	More than 60	6 (9%)	10 (15%)	50 (76%)	0.001	610.00
BMI	Normal	22 (68%)	6 (18%)	4 (14%)	0.012	400.00
	Preobese	16 (16.6%)	30 (31.4%)	50 (52%)	0.012	324.00
	Obese	28 (21.8%)	45 (35.2%)	55 (43%)	0.012	430.00
	Severe obese	4 (6.2%)	11 (17.1%)	49 (76.7%)	0.012	370.00
Duration of diabetes in years	1 to 2 years	40 (44.4%)	27 (30%)	23 (24.6%)	0.0021	320.00
	3 to 5 years	44 (26.8%)	50 (30.4%)	70 (42.6%)	0.0021	360.00
	More than 6 years	11 (16.6%)	15 (22.4%)	40 (61%)	0.0021	380.00

A similar pattern is observed with BMI. Individuals with normal BMI had the highest percentage of normal vitamin B12 levels (68%) and the lowest deficiency rate (14%). In contrast, those categorized as severely obese showed a deficiency rate of 76.7%, with only 6.2% having normal levels. The increasing deficiency with rising BMI (p=0.012) underscores the impact of obesity on vitamin B12 status.

Additionally, the duration of diabetes is also significantly associated with vitamin B12 levels. Among individuals with diabetes for one to two years, 44.4% had normal vitamin B12 levels, and only 24.6% were deficient. In contrast, those with a history of diabetes for more than six years showed a deficiency rate of 61%, with just 16.6% having normal levels (p=0.0021). These findings clearly demonstrate that the longer the duration of diabetes, the higher the risk of vitamin B12 deficiency.

In conclusion, the data shows a statistically significant relationship between increasing age, obesity, and duration of diabetes with rising prevalence of vitamin B12 deficiency. These results highlight the importance of regular screening for vitamin B12 levels, especially in older adults, individuals with higher BMI, and those with long-standing diabetes (Table 2).

**Figure 1:**
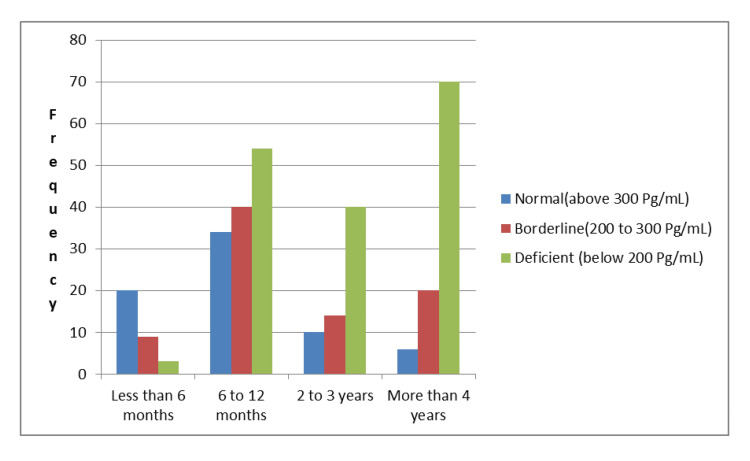
Association of metformin duration and dosage with vitamin B12 levels among patients

Table 3 presents the distribution of vitamin B12 levels among individuals based on the duration and dosage of metformin use. It shows a clear inverse relationship between both the duration and dose of metformin with vitamin B12 levels. Among those using metformin for less than six months, the majority (62.5%) had normal vitamin B12 levels, while only 9.4% were found to be deficient. As the duration of metformin use increased, the proportion of individuals with vitamin B12 deficiency also rose significantly. For instance, among those using metformin for two to three years, 62.5% were deficient, and this increased to 73% among those using it for more than four years. Similarly, the current dose of metformin also showed a significant impact on vitamin B12 status. At a lower dose of 500 mg/day, half of the participants (50%) had normal vitamin B12 levels, and only 18.8% were deficient. However, as the dose increased to 1000 mg/day, the proportion of deficient individuals rose to 62.5%, and at doses between 1000 and 2000 mg/day, 78.2% of participants were vitamin B12 deficient. The chi-square values (434.286 for duration and 80.00 for dose) and p-values (<0.001) indicate a highly significant association between both variables and vitamin B12 deficiency, suggesting that longer duration and higher doses of metformin are strongly linked with increased risk of vitamin B12 deficiency (Table 3 and Figure 1).

**Table 3:** Association of metformin duration and dosage with vitamin B12 levels among patients

Variables	Category	Vitamin B12 level			P-value	Chi-square value
		Normal (above 300 Pg/mL), N (%)	Borderline (200 to 300 Pg/mL), N (%)	Deficient (below 200 Pg/mL), N (%)		
Duration of metformin use in months/years	Less than 6 months	20 (62.5%)	9 (28.12%)	3 (9.4%)	0.001	434.286
	6 to 12 months	34 (26.5%)	40 (31.25%)	54 (42.3%)	0.001	334.490
	2 to 3 years	10 (15.6%)	14 (21.8%)	40 (62.5%)	0.001	234.116
	More than 4 years	6 (6.2%)	20 (20.8%)	70 (73%)	0.001	634.186
Current metformin dose in mg/dL	500 mg/dL	64 (50%)	40 (31.2%)	24 (18.8%)	0.001	90.00
	1000 mg/dL	16 (12.5%)	32 (25%)	80 (62.5%)	0.001	75.00
	More than 1000 to 2000 mg/dL	4 (6.2%)	10 (15.6%)	50 (78.2%)	0.001	50.00

## Discussion

In comparison to the findings of the referenced study, where a significantly higher prevalence of vitamin B12 deficiency was observed among metformin users (38.76%) compared to non-users (21.42%) [13], our study revealed an even more pronounced deficiency rate of 65.7% among metformin users. This suggests a potentially stronger association in our population, possibly due to factors such as higher average BMI, older age distribution, or longer duration and higher doses of metformin use. The referenced study further highlights that the highest deficiency rate (111.00%) was found in individuals using metformin for more than 15 years, indicating a cumulative effect of prolonged metformin therapy. Although our study did not include a group with more than 15 years of metformin use, we similarly found a significantly rising trend in deficiency with longer duration; 73% of those using metformin for more than four years were deficient. Moreover, the association between metformin use and vitamin B12 deficiency in the referenced study was statistically significant, with an odds ratio of 7.17 (p<0.001), which supports our findings, where chi-square tests also revealed highly significant associations (p=0.001) between both duration and dosage of metformin with B12 deficiency.

In contrast to the study conducted in Bangladesh, which reported a 31.1% prevalence of vitamin B12 deficiency and 6.7% borderline deficiency among 90 subjects [14], our study demonstrated a significantly higher prevalence of B12 deficiency, affecting 65.7% of metformin users. This difference may be attributed to variations in sample size, population characteristics, dietary patterns, or healthcare access between the two regions. In the Bangladeshi study, subjects with subnormal B12 levels had a notably longer duration of metformin use, with a median of 8.5 years compared to five years in those with normal levels (p=0.006), suggesting a clear association between prolonged metformin use and B12 deficiency. Our findings support this trend, as we also observed a significantly higher rate of deficiency (73%) among those using metformin for more than four years. Moreover, the gram-years of metformin use were substantially higher in the deficient group in the Bangladesh study (12.0 vs. 5.75), aligning with our results, where higher daily doses of metformin were significantly associated with B12 deficiency (p=0.001). Both studies reinforce the dose- and duration-dependent impact of metformin on vitamin B12 levels, highlighting the need for timely screening and possible supplementation in long-term users.

Compared to the study conducted in Nepal, which reported a 50.95% prevalence of vitamin B12 deficiency among 210 patients, 107 with severe deficiency and 63 with borderline levels [15], our findings reveal an even higher prevalence of deficiency among metformin users (38.76%) and a borderline level in an additional 9.1%. Similar to the Nepalese study, our results demonstrated that the mean vitamin B12 level decreased significantly with increasing duration of metformin use. In both studies, longer metformin use was clearly associated with a higher prevalence of deficiency, reinforcing the duration-dependent effect of the drug on B12 levels. Moreover, both studies found no significant association between sex or age and the development of B12 deficiency, suggesting that these demographic factors may not play a substantial role in the risk. Notably, in our study, the highest prevalence (11.1%) of B12 deficiency was observed in patients using metformin for more than 15 years, and the association between metformin use and B12 deficiency was statistically significant (OR=7.17; 95% CI: 2.46-20.92; p<0.001). This further supports the findings from Nepal and emphasizes the clinical importance of monitoring vitamin B12 levels in patients on long-term metformin therapy.

In comparison to the Libyan study, which reported a 23.84% prevalence of vitamin B12 deficiency among metformin users and 14.28% in the control group, our study found a notably higher prevalence of B12 deficiency among metformin users at 38.76% and only 21.42% among non-users [16]. This highlights a stronger association between metformin use and vitamin B12 deficiency in our population.

The Libyan study also showed that the mean serum B12 level in the metformin group (443.65 ± 227.34 pg/mL) was significantly lower than that of the control group (541.44 ± 283.65 pg/mL), with a statistically significant p-value of 0.003. Similarly, in our study, we observed a significant association between metformin use and B12 deficiency (OR=7.17; 95% CI: 2.46-20.92; p<0.001), reinforcing the impact of metformin on reducing B12 levels.

While the Libyan study noted that borderline B12 deficiency was most common in individuals aged 30 to 50 years (23.15%), our findings did not identify age as a significant factor influencing B12 deficiency. This difference suggests that age-specific trends may vary by population or sample characteristics.

Overall, both studies support the association between metformin use and reduced serum vitamin B12 levels, with our study indicating a higher magnitude of deficiency and stronger statistical association.

Limitations

This study, while providing important insights into the prevalence of vitamin B12 deficiency among patients with T2DM on long-term metformin therapy, had certain limitations. First, it was a cross-sectional study conducted in tertiary care hospitals of Peshawar, which may limit the generalizability of the findings to the broader population, particularly rural or primary care settings. Second, dietary intake of vitamin B12 and the use of vitamin supplements were not assessed, which could have influenced serum vitamin B12 levels and acted as confounding factors. Additionally, the study did not evaluate the clinical symptoms or neurological manifestations of vitamin B12 deficiency, which would have added valuable information regarding the functional impact of low vitamin B12 levels. The reliance on a single serum B12 measurement without further confirmatory tests like methylmalonic acid (MMA) or homocysteine levels may also limit the accuracy of deficiency diagnosis. Finally, due to resource and time constraints, a longitudinal follow-up was not possible, which would have helped assess the progression or reversal of deficiency over time with intervention.

## Conclusions

This study reveals a high prevalence of vitamin B12 deficiency among T2DM patients on long-term metformin therapy in tertiary care hospitals of Peshawar, with 65.7% deficient and 24.3% showing borderline levels. Statistically significant associations were found between B12 deficiency and older age, higher BMI, longer diabetes duration, and extended metformin use, particularly in patients over 60 years, severely obese individuals, and those using metformin for over four years. These findings align with existing literature, underscoring the need for routine B12 screening in T2DM patients on prolonged metformin therapy to prevent complications like anemia, neuropathy, and cognitive decline, conditions often misattributed to diabetes itself. Early detection and supplementation are essential for improving outcomes, and further studies are recommended to develop standardized monitoring guidelines.

## Appendices

Section A: Demographic Information

Name (Initials only): ___________

Age: ________

Gender: ☐ Male ☐ Female

Height (cm): ________

Weight (kg): ________

BMI (calculated): ________

Occupation: ___________

Education Level: ☐ No formal education ☐ Primary ☐ Secondary ☐ Graduate ☐ Postgraduate

Section B: Clinical History

Duration of Diabetes: ________ years

Current Anti-diabetic Medications: ☐ Metformin ☐ Insulin ☐ Sulfonylureas ☐ DPP-4 Inhibitors ☐ Others (specify): ___________

Duration of Metformin Use: ________ months/years

Current Metformin Dose: ________ mg/day

Any previous B12 test? ☐ Yes ☐ No

Any B12 supplementation in the past 6 months? ☐ Yes ☐ No

Are you a strict vegetarian? ☐ Yes ☐ No

Any history of gastric/bariatric surgery? ☐ Yes ☐ No

Do you consume alcohol? ☐ Yes ☐ No

Do you take Proton Pump Inhibitors or H2 blockers regularly? ☐ Yes ☐ No

**Section C: Symptom Assessment** Have you experienced any of the following recently (past 3 months)? (☐ Yes ☐ No for each)

Numbness or tingling in hands/feet ☐

Burning sensation in feet ☐

Difficulty walking or unsteady gait ☐

Fatigue or weakness ☐

Memory issues or confusion ☐

Glossitis or soreness of tongue ☐

Shortness of breath on mild exertion ☐

Section D: Lab Investigations (To be filled after testing)

Serum Vitamin B12 level (pg/mL): ________

Fasting Blood Sugar (optional): ________

HbA1c (optional): ________
